# Tumor promoting effects of CD95 signaling in chemoresistant cells

**DOI:** 10.1186/1476-4598-9-161

**Published:** 2010-06-23

**Authors:** Elisabet Ametller, Susana García-Recio, Domizziana Costamagna, Cristina Mayordomo, Patricia Fernández-Nogueira, Neus Carbó, Eva María Pastor-Arroyo, Pedro Gascón, Vanessa Almendro

**Affiliations:** 1 Medical Oncology, Institut d'Investigacions Biomèdiques Agustí Pi y Sunyer (IDIBAPS), Institut Clínic de Malalties Hemato-Oncològiques (ICMHO), Hospital Clínic, Facultat de Medicina, Universitat de Barcelona, Spain; 2 Dipartimento di Medicina e Oncologia Sperimentale, Università di Torino, Italy; 3 Departament de Bioquímica i Biologia Molecular, Facultat de Biologia and Institut de Biomedicina (IBUB), Universitat de Barcelona, Spain

## Abstract

**Background:**

CD95 is a death receptor controlling not only apoptotic pathways but also activating mechanisms promoting tumor growth. During the acquisition of chemoresistance to oxaliplatin there is a progressive loss of CD95 expression in colon cancer cells and a decreased ability of this receptor to induce cell death. The aim of this study was to characterize some key cellular responses controlled by CD95 signaling in oxaliplatin-resistant colon cancer cells.

**Results:**

We show that CD95 triggering results in an increased metastatic ability in resistant cells. Moreover, oxaliplatin treatment itself stimulates cell migration and decreases cell adhesion through CD95 activation, since CD95 expression inhibition by siRNA blocks the promigratory effects of oxaliplatin. These promigratory effects are related to the epithelia-to-mesenchymal transition (EMT) phenomenon, as evidenced by the up-regulation of some transcription factors and mesenchymal markers both *in vitro *and *in vivo*.

**Conclusions:**

We conclude that oxaliplatin treatment in cells that have acquired resistance to oxaliplatin-induced apoptosis results in tumor-promoting effects through the activation of CD95 signaling and by inducing EMT, all these events jointly contributing to a metastatic phenotype.

## Background

CD95 (APO-1/Fas), a 48 kDa membrane protein belonging to the TNF receptor superfamily, activates caspase-dependent apoptosis in susceptible cells when is activated by its natural ligand (CD95L). There are two different cell types that use distinct CD95 apoptosis signaling pathways. After CD95 triggering, type I cells activate caspase-8 at the death-inducing signaling complex (DISC) followed by activation of caspase-3. Apoptosis induction in these cells is not sensitive to mitochondria pathway inhibition. However, in type II cells DISC formation is strongly reduced and the activation of caspase-8 and caspase-3 is produced downstream of mitochondrial events. Thus, inhibition of the mitochondrial role in these cells by overexpression of Bcl-2 or Bcl-x_L _blocks apoptosis [1,2]. This classification is related to cell phenotype, since type I cells correspond to mesenchymal tumors and type II cells display a more epithelial phenotype [3].

Many cancer cells acquire survival advantage during tumor progression by decreasing its sensitivity to CD95-induced apoptosis [4,5]. Some mechanisms affecting CD95 sensitivity include downregulation of CD95 protein expression [6], blocking of the active receptor site by the soluble form of CD95 ligand (sCD95L) [7], blocking of the interaction of CD95L with CD95 by the soluble decoy receptor 3 [8] and altered survival signaling pathways, among others (reviewed in [9]). In fact, this loss of CD95-responsiveness seems to be produced in parallel to tumor progression towards a more metastatic phenotype [5,10]. In colon cancer cell lines the functional elimination of CD95-responsive cells select for the outgrowth of different metastatic subpopulations, and cells isolated from the metastatic sites of xenografts tumors are resistant to CD95-induced apoptosis [10]. In agreement with this observation, there is almost a complete loss of detectable CD95 expression in metastatic lesions of colon cancer patients compared with the primary lesions [11].

However, this loss of sensitivity to CD95-induced apoptosis could reflect a change in its functionality since some of these CD95 apoptosis-resistant cells respond to CD95 activation with increased motility and invasiveness [5,12], contributing to the development of the metastatic phenotype. In fact, it has been recently demonstrated that human and mouse colon cancer cells metastasize to the liver by using CD95 signaling [13]. Therefore, CD95 receptor can also exert roles beyond apoptosis and favor tumor-promoting effects in CD95 apoptosis-resistant tumor cells [12]. The selective pressure exerted by oxaliplatin treatment also selects for apoptosis-resistant cells with alterations in CD95-regulated signaling pathways and decreased CD95 expression. In these oxaliplatin-resistant cells, activation of CD95 induces the phosphorylation of p42/44 MAPK and p38 MAPK, suggesting that changes in the receptor functionality are related to the acquisition of resistance to oxaliplatin [14]. In fact, induction of cell death by some cytotoxic drugs seems to be cell-type specific and to depend on the presence of an intact CD95 system [15]. For example, the apoptotic-effects of some cytotoxic agents such as doxorubicin and oxaliplatin are mediated by upregulation of CD95 and CD95L expression, and by activation of the apoptotic signaling in the neighboring cells [7]. Therefore, tumor progression or mechanisms of selective pressure that alter CD95 status would subsequently affect chemotherapy sensibility and cell behavior.

We previously reported that, in a way similar to what happens during tumor progression, acquisition of chemoresistance also selects for cells in which the functional activation of CD95 does not induced apoptosis but instead activates MAPK proteins and the NF-κB pathway [14]. Since oxaliplatin treatment is known to increase CD95 expression in both sensitive and resistant cell lines [14] the aim of the present study was to ascertain if oxaliplatin up-regulation of CD95 expression could contribute to the acquisition of a more aggressive behavior. Briefly, we show that drug treatment stimulates migration and decreases adhesion by means of CD95 activation in cells that have acquired resistance to oxaliplatin-induced death. We also report that oxaliplatin-resistant cells undergo EMT, as evidenced by the expression profile of several markers, and that oxaliplatin treatment also contributes to the acquisition of a more mesenchymal phenotype in the resistant cells and tumor xenografts. Finally, we have found that CD95 triggering in chemoresistant cells activates MAPK pathways and alters the expression levels of certain cell cycle proteins with previously described promigratory roles. We conclude that oxaliplatin treatment in cells that have acquired resistance to oxaliplatin-induced apoptosis could result in tumor-promoting effects by activation of CD95 promigratory signaling and by inducing EMT, which jointly contribute to the acquisition of a more metastatic phenotype.

## Results and Discussion

### Oxaliplatin induces cell migration by activation of CD95 receptor in resistant cells

Some colon cancer cell lines resistant to CD95-induced apoptosis respond to CD95 activation with increased motility and invasiveness. Taking into account that oxaliplatin treatment increases CD95 expression in both sensitive and resistant cell lines, we aimed to determine if oxaliplatin stimulation of CD95 expression could exert a pro-migratory effect. To address directly this question we used a previously characterized model of acquired chemoresistance based on the cell lines HT29 and HCT116 p53^-/- ^and their corresponding oxaliplatin-resistant derived cell lines RHT29, and RHCT116 p53^-/- ^[14]. To determine the effects of oxaliplatin and CD95 activation on cell migration we performed transwell migration assays as described in the Material and Methods section.

**Figure 1:**
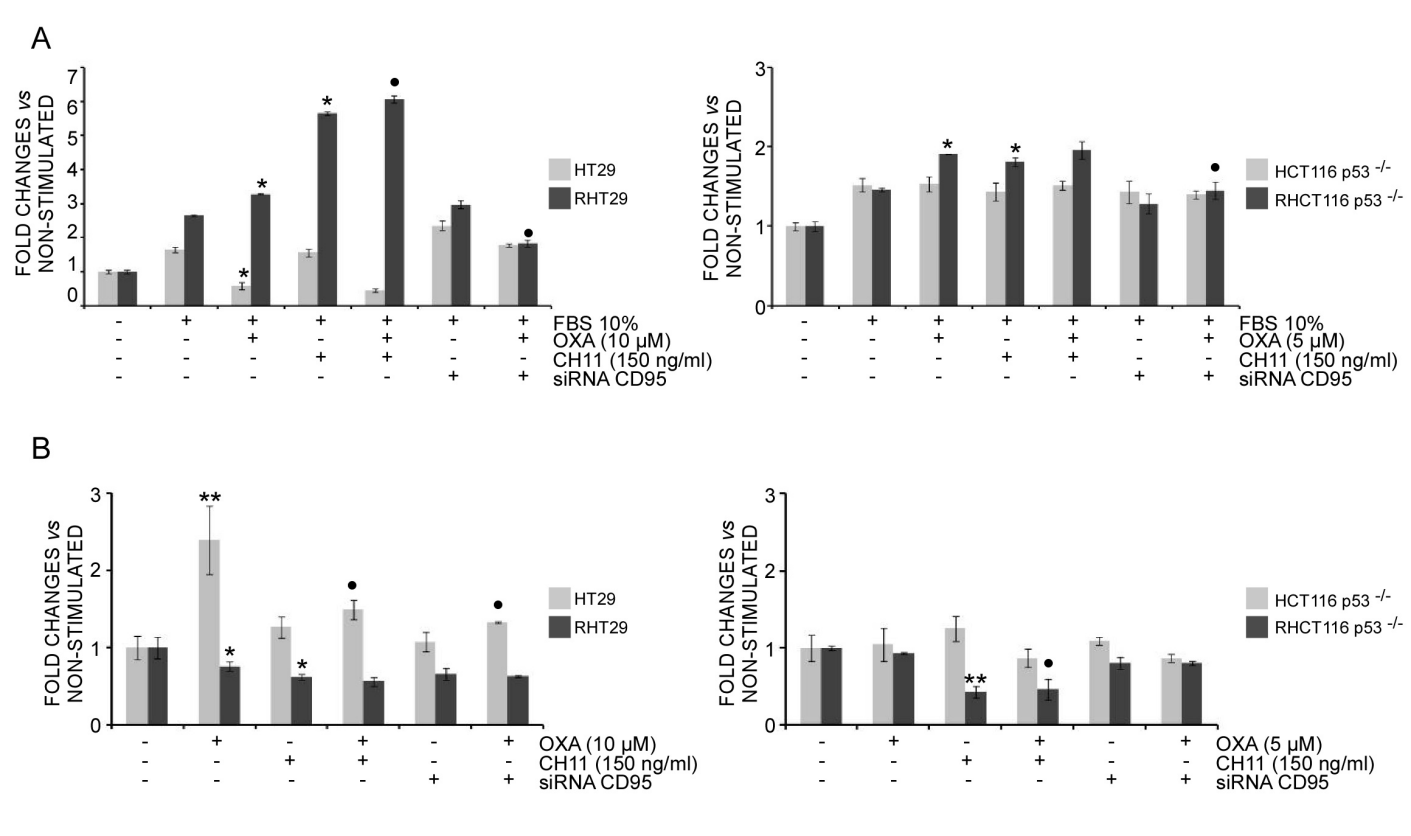
**Effects of oxaliplatin and CD95 activation on cell migration and adhesion in oxaliplatin-resistant cells**. **A) **To determine the effects of oxaliplatin or CD95 triggering on cell migration different sensitive (HT29 and HCT116 p53^-/-^) and resistant (RHT29 and RHCT116 p53^-/-^) cell lines were treated with oxaliplatin (10 μM for the HT29 and RHT29 and 5 μM for the HCT116 p53^-/- ^and RHTC116 p53^-/-^) or/and the CD95 agonistic antibody CH11 (150 ng/ml) for 24 hours, and their migratory ability was assessed in transwell assays. The contribution of CD95 activation to oxaliplatin-induced cell migration was determined by inhibiting CD95 expression with siRNA. **B) **The effects of oxaliplatin or CD95 triggering on the adhesion ability of the different cell lines or the contribution of CD95 activation to oxaliplatin-induced cell adhesion were determined under the same experimental conditions on fibronectin coated plates. The experiments were performed in triplicate and results represent the mean ± SEM. Values that are significantly different from control group by ANOVA's analysis are indicated by *p < 0.05, **p < 0.01, and those different from the oxaliplatin-treated group are indicated by •p < 0.05. *OXA*: oxaliplatin, *FBS*: Fetal bovine serum.

As expected, FBS stimulation induced a significant increase in basal cell migration both in HT29 and RHT29 cells (1,7 and 1,9-fold induction, respectively) and in the HCT116 p53^-/- ^and RHCT116 p53^-/- ^(1,5-fold induction in both cell lines) (Figure 1A). Pretreatment of cells with oxaliplatin results in a higher migration capacity in RHT29 and RHCT116 p53^-/- ^cells (1,3-fold induction in both cases). Interestingly, a strong stimulation of cell migration was observed again in RHT29 and RHCT116 p53^-/- ^cell lines (3-fold and 1,5-fold induction, respectively) after agonistic CH11 antibody treatment (Figure 1A). This effect was not significantly enhanced when cells were pre-treated with oxaliplatin, probably due to the promigratory effects exerted by CH11 alone that masked the increase induced by oxaliplatin. On the other hand, neither HT29 nor HCT116 p53^-/- ^cells were affected by CH11-induced CD95 activation or drug pretreatment (Figure 1A), indicating that the activation of CD95 only induces cell migration in the oxaliplatin-resistant cell lines.

To better understand the contribution of CD95 in oxaliplatin-induced cell migration CD95 expression was abrogated by siRNA under the same experimental conditions (Figure 1A and Additional file 1: Fig S1). The inhibition of CD95 expression did not affect basal migration rate, but completely abrogated oxaliplatin-induced migration in the RHT29 and RHCT116 p53^-/- ^cell lines, confirming that the effects of oxaliplatin pretreatment in cell migration are mediated by the activation of CD95 receptor. Similar results were obtained when the cells were treated with the CD95-blocking antibody DX2 (Additional file 2: Fig S2). Finally, cell growth curves were performed under the same experimental conditions and no differences in cell proliferation rates were found (data not shown), ruling out any possible contribution of differences in proliferation rates among cell lines to the migration assay results.

To determine if these effects on cell migration were accompanied by changes on cell adhesion we determined the capability of the various cell lines to adhere to fibronectin-coated surfaces. Oxaliplatin pretreatment significantly increased cell adhesion in HT29 cells but not in RHT29 cells (Figure 1B). Interestingly, however, CD95 stimulation by CH11 induced a significant decrease on basal adhesion ability in the RHT29 cell line and this was also evident even under oxaliplatin-pretreatment. CD95 inhibition by siRNA slightly affected RHT29 adhesion ability and significantly increased basal HT29 adhesion under oxaliplatin-pretreatment. Similar results were obtained when CD95 activation was blocked with DX2 antibody (Additional file 2: Fig S2). Finally, CD95 activation by CH11 also decreased cell adhesion in the RHCT116 p53^-/- ^cell line, while remaining unaltered by any treatment in HCT116 p53^-/- ^cells (Figure 1B).

Taken together, these results indicate that oxaliplatin treatment stimulated cell migration in oxalipaltin-resistant cell lines by activating CD95 receptor, since forced activation of CD95 by an agonistic antibody strongly increased migration and decreased cell adhesion while CD95 silencing and inhibition efficiently abrogated oxaliplatin effects.

### Treatment with oxaliplatin induces the phosphorylation of p42/44 MAPK and p38 MAPK proteins

Stimulation of motility and invasiveness by CD95 is mediated by the activation of different signaling pathways including caspase-8, NF-κB and phospho-p42/44 MAPK [12]. Moreover, oxaliplatin induces the activation of p38 MAPK [16] and NF-κB [14,17] in several cell lines. The p38 MAPK has been described as a classical tumor suppressor [18,19], but p38 MAPK seems to play also other roles, such as increasing cell survival as a cellular defense mechanism to overcome the effects of cytotoxic drugs and also promoting cellular migration [20]. In addition, oxaliplatin increases NF-κB signaling in both HT29 and RHT29 cells but only the RHT29 cell line has dependence on the NF-κB pathway for survival [14]. For these reasons we next decided to determine if oxaliplatin treatment differentially contributes to the activation of p42/44 MAPK, p38 MAPK and the p65 subunit of NF-κB in the HT29 and RHT29 cell lines. We also investigated the signaling pathways activated by CD95 under oxaliplatin treatment.

**Figure 2:**
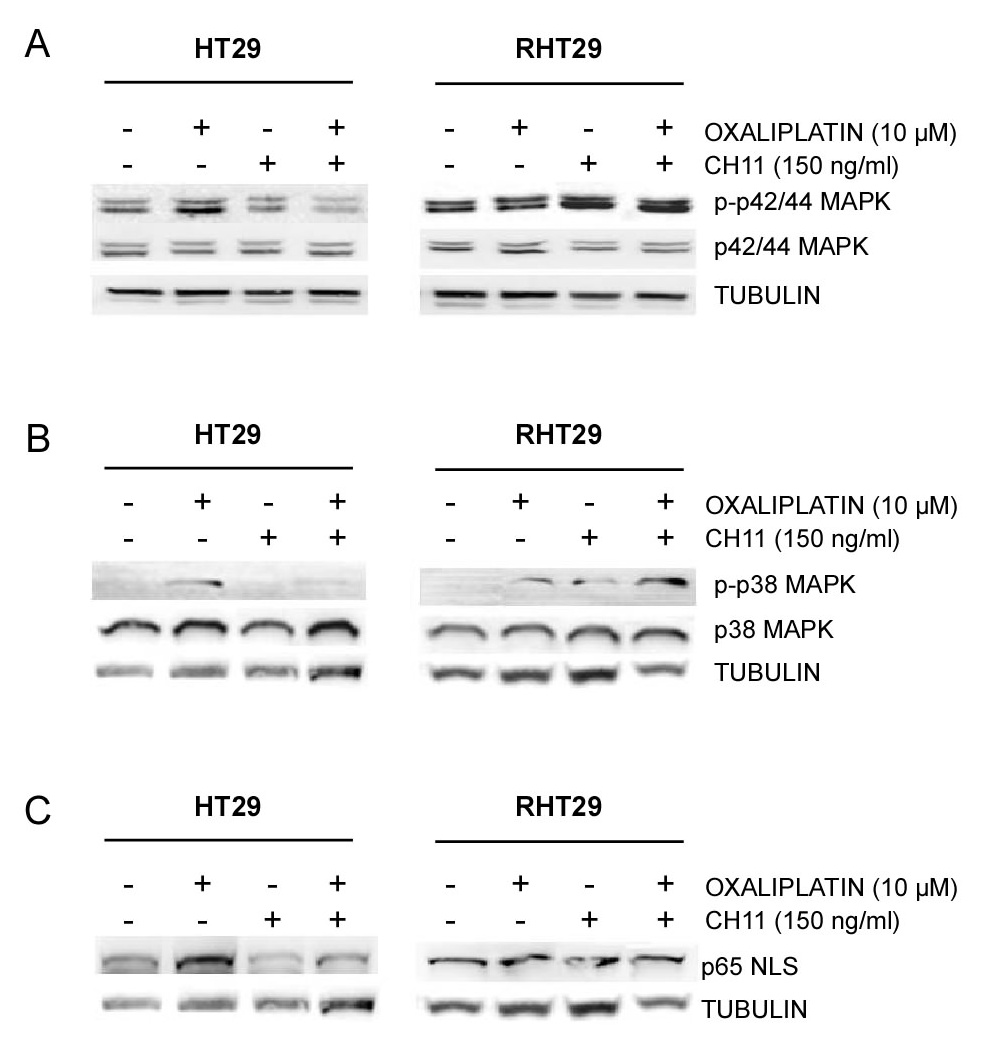
**Differential activation of MAPK pathways by CD95 in cells resistant to oxaliplatin-induced cell death**. To determine the contribution of oxaliplatin-induced CD95 to the activation of **A) **p42/44 MAPK, **B) **p38 MAPK signaling pathways and to the activation of **C) **NF-κB (p65 NLS), cells were treated with oxaliplatin (10 μM), the CD95 agonistic antibody CH11 (150 ng/ml) or the combination of both for 48 hours, and the protein levels determined by Western blot.

HT29 and RHT29 cells were treated with oxaliplatin 10 μM for 48 hours and with 150 ng/ml of the CD95 agonistic antibody CH11 for 24 hours. As previously observed, oxaliplatin treatment activated p42/44 MAPK (Figure 2A) and p38 MAPK (Figure 2B). Activation of CD95 by CH11 increased the levels of p42/44 MAPK (Figure 2A) and p38 MAPK (Figure 2B) in the oxaliplatin-resistant cell line, but not in the sensible one. Moreover, oxaliplatin treatment slightly increased the effects of the agonistic antibody CH11 (Figure 2A and 2B). The inhibition of extracellular metalloproteinases, which decreased CD95 expression by receptor cleavage at the plasma membrane, also enhanced the effects of CH11 (data not shown), confirming the involvement of CD95 in the activation of MAPK pathways in the RHT29 cell line. Total amounts of active NF-κB were not significantly increased by CD95 activation in any of the cell lines analyzed (Figure 2C).

These results suggest that in cells that have acquired resistance to oxaliplatin CD95 triggering stimulates MAPK pathways known to contribute to the motility and invasiveness of these cells.

### Oxaliplatin treatment differentially affects cell cycle and apoptosis-related proteins

NF-κB is constitutively expressed in a wide range of tumor cells, where it commonly promotes cell proliferation and resistance to apoptosis induced by genotoxic agents [17,21]. After exposure to oxaliplatin cells can suffer an arrest in G1 or G2/M followed by an S-phase delay [22-24]. Taking into account that cells respond to oxaliplatin by increasing NF-κB activity [14], which controls cyclin D1 expression [25], we also decided to analyze if oxaliplatin treatment was differentially affecting cell cycle profiles, proteins levels and apoptosis related proteins.

**Figure 3:**
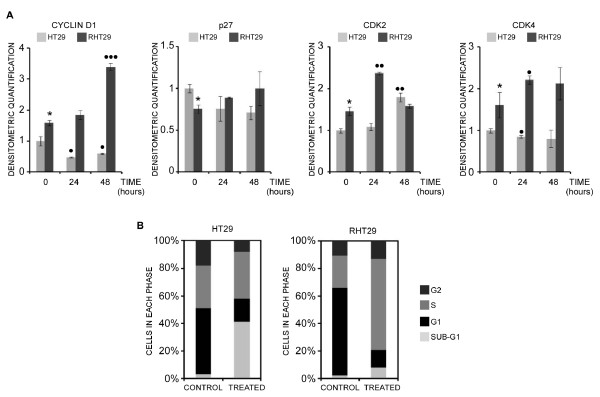
**Oxaliplatin regulation of cell cycle-related proteins in the HT29 and RHT29 cell lines**. **A**) HT29 and RHT29 cell lines were treated with oxaliplatin 10 μM for 24 and 48 hours and the expression of the cell-cycle related proteins cyclin D1, p27, CDK2 and CDK4 determined by Western blot. Results shown are the mean ± SEM of the densitometric quantification three independent experiments. Values that are significantly different between groups by ANOVA's analysis are indicated by *p < 0.05, and those different between non-treated and treated groups are indicated by •p < 0.05, ••p < 0.01, •••p < 0.001. **B**) Cell cycle studies: the distribution of the different phases of the cell cycle was determined in HT29 and RHT29 cells by flow cytometry. *CDK2*: cyclin-dependent kinase 2, *CDK4*: cyclin-dependent kinase 4.

HT29 and RHT29 cell lines were treated with oxaliplatin for 24 and 48 hours and protein expression was determined by Western blot analysis. Basal levels of cyclin D1 were higher in the RHT29 cells compared to the HT29 cells (Figure 3A). Oxaliplatin treatment induced opposing effects in these cell lines. Whereas a significant increase in cyclin D1 expression was found in the RHT29 cell line, parental cells showed lower cyclin D1 protein expression after oxaliplatin. Interestingly, the upregulation of cyclin D1 expression in the RHT29 cells agrees with the increase in active NF-κB previously observed [14] and might be supporting their increased migratory ability described in the present paper (Figure 1A). Basal expression of p27 was slightly lower in the RHT29 cell line, being not significantly affected by oxaliplatin treatment in any cell line.

Basal expression of CDK2 and CDK4 were significantly higher in the RHT29 cell line and oxaliplatin treatment increased CDK2 (transiently) and CDK4 expression (Figure 3A). CDK2 levels were found increased in HT29 cells after 48 h oxaliplatin treatment and a slight decrease was observed in CDK4 levels after 24 h of treatment in the HT29 cell line. Therefore, the RHT29 cell line showed higher levels of proteins related to proliferation and cell cycle progression and lower levels of the cell cycle inhibitor p27, all these changes accounting for its acquired resistance to oxaliplatin.

Next, we analyzed by flow cytometry the effects of 48 hours of oxaliplatin exposure (10 μM) in the cell cycle distribution of both cell lines. The percentage of cells in the sub-G1 phase was significantly higher in the HT29 cell line compared to the RHT29 cell line, as expected (Figure 3B). In this case no arrest in the G1 phase was detected, probably because the dose and the time analyzed induced the majority of cells to undergo cell death. However, oxaliplatin induced an S-phase cell accumulation in the RHT29 cell line, suggesting that the drug could be promoting G1/S transition by upregulating cyclin D1, CDK4 and CDK2 but making it difficult to complete the S-phase, thus exerting a cytostatic rather than a cytotoxic effect on these oxaliplatin-resistant cells (Figure 3B).

**Figure 4:**
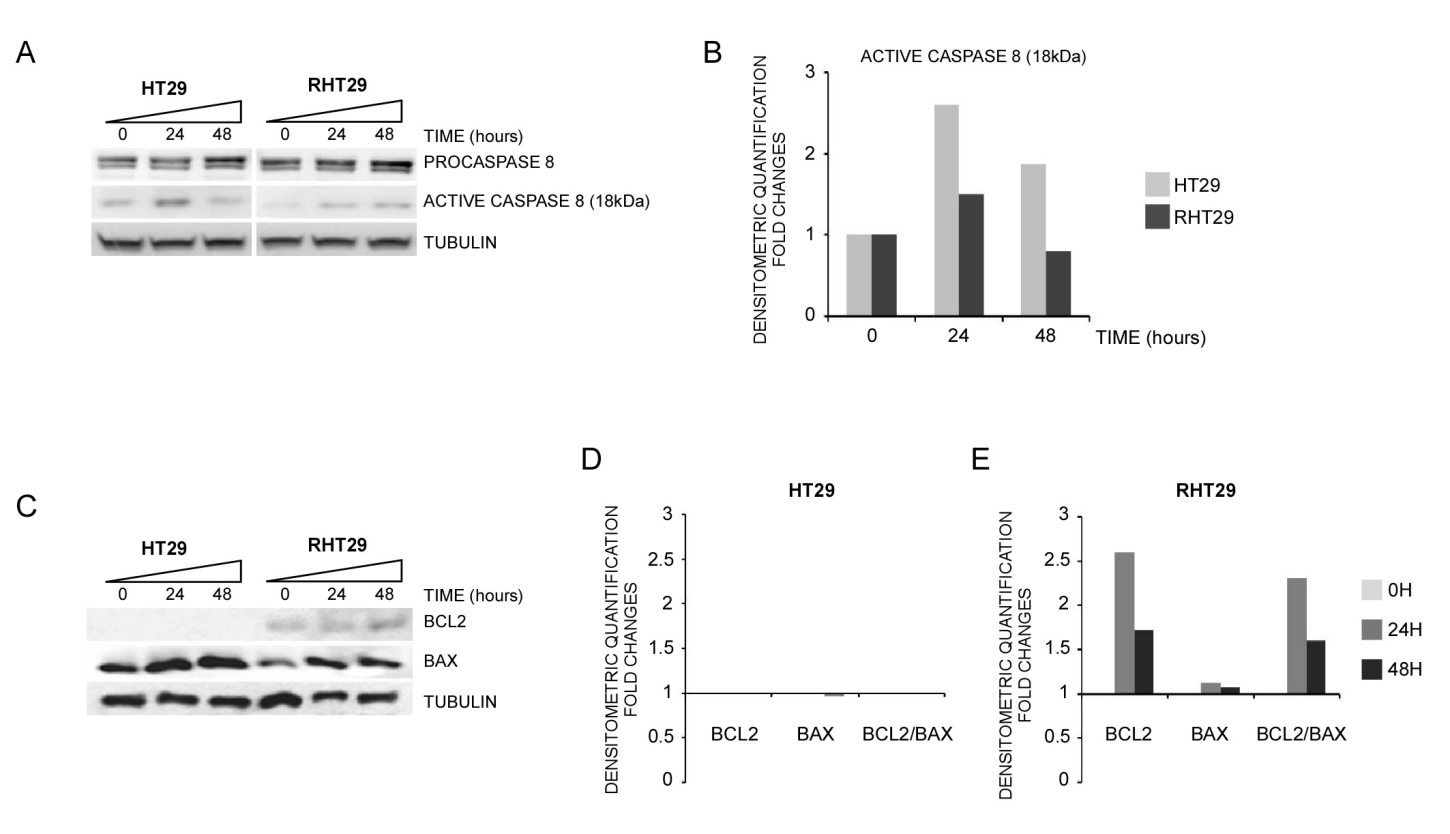
**Oxaliplatin regulation of apoptosis-related proteins in the HT29 and RHT29 cell lines**. HT29 and RHT29 cell lines were treated with oxaliplatin 10 μM for 24 and 48 hours and the expression of the **A**) active caspase 8 and **C**) Bcl-2 and Bax were determined by Western blot. **B**) Densitometry of active caspase 8. **D-E**) Ratio between Bcl-2 and Bax for the HT29 and RHT29 cell lines based on the densitometry quantification.

We also determined the expression levels of active caspase-8, Bcl-2 and Bax proteins in HT29 and RHT29. We found that in the resistant cell line RHT29 oxaliplatin was less effective in inducing the activation of caspase-8 (Figure 4A and 4B). Furthermore, we also found increased basal Bcl2 levels and an increased Bcl2/Bax ratio in the oxaliplatin-treated RHT29 cells (Figure 4C-4E) further indicating that in this resistant cell line, the upregulation of survival pathways is contributing to the resistance to oxaliplatin-induced apoptosis.

### Acquisition of resistance to oxaliplatin induces EMT and confers a more motile phenotype

**Figure 5:**
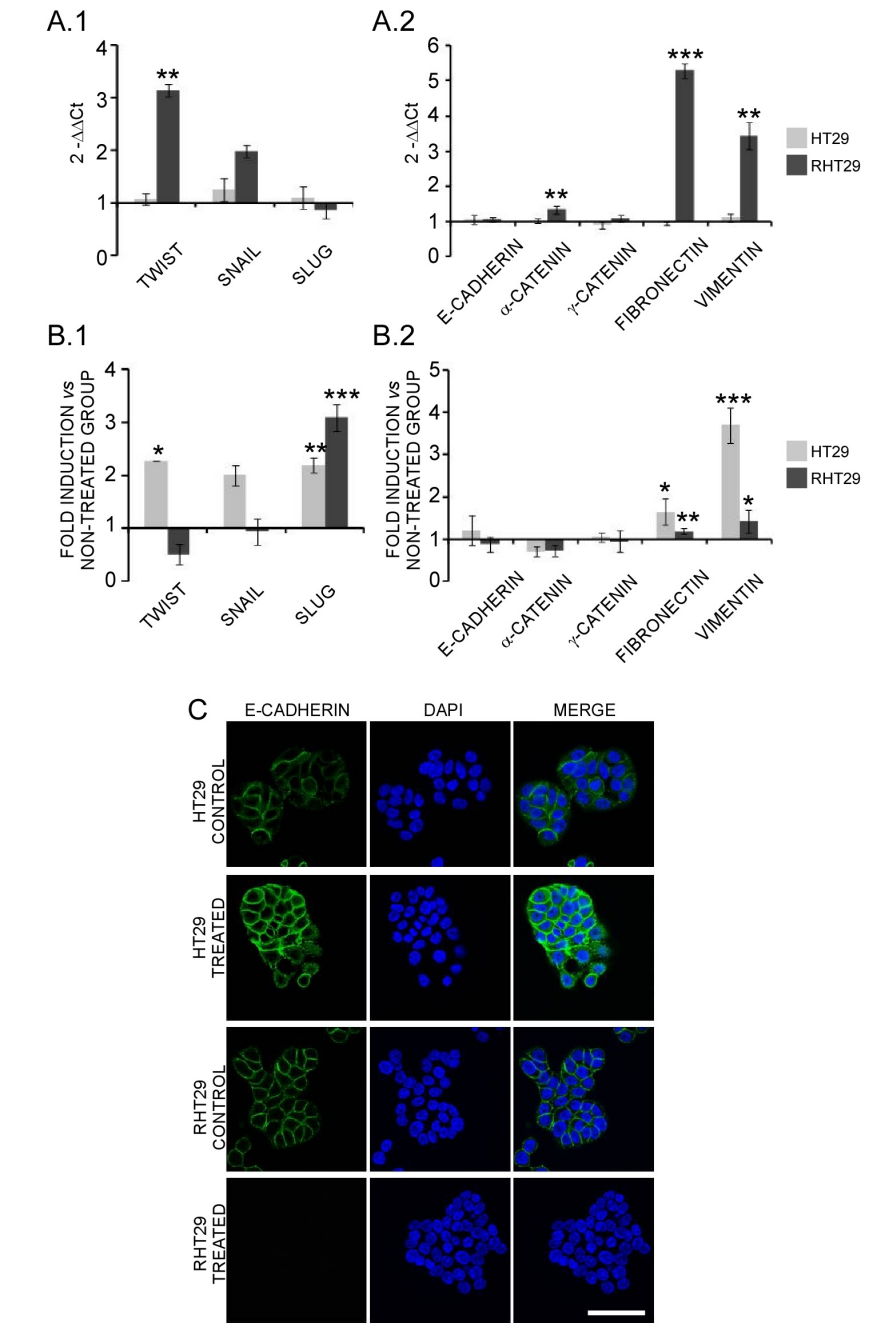
**Analysis of EMT markers and E-cadherin expression on HT29 and RHT29 cells**. **(A1) **Differential basal expression of some transcription factors related to the EMT process and **(A2) **markers of epithelial and mesenchymal phenotype were determined in the HT29 and RHT29 cell lines by qPCR. (**B1**) The effects of oxaliplatin in the gene expression of those transcription factors and (**B2**) EMT markers were also analyzed in cells treated with oxaliplatin 10 μM for 48 hours and in this case the results are shown as fold induction *versus *non-treated cells. Results represent the mean ± SEM of triplicates. Values that are significantly different between (**A**) groups or between (**B**) non-treated and treated groups by ANOVA's analysis are indicated by *p < 0.05, **p < 0.01, ***p < 0.001. **C) **HT29 and RHT29 cells were treated with oxaliplatin 10 μM for 48 hours and E-cadherin expression was detected by immunofluorescence and confocal microscope analysis. Scale bar is 50 μm.

The differential cell migratory responses of sensitive and resistant colon cancer cells to oxaliplatin led us to consider the possibility that resistant cells have undergone epithelial-to-mesenchymal transition (EMT), as described by others [26] and thus their motility capacity could result concomitantly affected. Gene expression of epithelial and mesenchymal markers and transcription factors related to the EMT reprogramming was studied under basal and oxaliplatin-treated conditions to determine if they were differentially expressed in HT29 and RHT29 cells. Figures 5 A1 and 5 A2 show the differential basal expression of the EMT markers between HT29 and RHT29 cells. In spite of the lack of any significant decrease in the epithelial markers E-cadherin, α-catenin or γ-catenin, basal expression of the transcription factors Twist and Snail and the mesenchymal markers fibronectin and vimentin were significantly higher in the oxaliplatin-resistant cell line RHT29 (Figures 5 A1 and 5 A2), suggesting that parallel to the acquisition of chemoresistance there is a change in the expression profile of these markers towards a more mesenchymal phenotype [26]. Figures 5 B1 and 5 B2 show the differential induction of the same markers under oxaliplatin treatment in both cell lines, expressed as fold-induction *versus *basal levels. Although oxaliplatin failed to induce cell migration in the HT29 cell line, drug treatment up-regulated the expression of the transcription factors Twist, Snail and Slug, decreased α-catenin expression and increased the mesenchymal markers fibronectin and vimentin (Figures 5 B1 and 5 B2). In the RHT29 cell line oxaliplatin seems to enhance the mesenchymal phenotype increasing the expression of some of the EMT markers such as Slug, fibronectin and vimentin and decreasing α-catenin (Figures 5 B1 and 5 B2). In agreement with the stimulation of the EMT process, oxaliplatin treatment (10 μM for 24 hours) induced the development of a more fibroblast-like, spindle-cell morphology in both cell lines (Additional file 3: Fig S3).

We also determined the expression of E-cadherin by immunofluorescence and confocal microscopy analysis (Figure 5C). Interestingly, E-cadherin levels increased in the plasma membrane of the HT29 cells treated with oxaliplatin, in agreement with the slight increase on mRNA levels observed by qPCR. This could be related to the increased adhesion of the HT29 cells observed under oxaliplatin treatment (Figure 1B). Instead, basal E-cadherin expression progressively decreased in oxaliplatin-treated RHT29 cells (data not shown), being completely abrogated after 30 minutes (Figure 5C).

**Figure 6:**
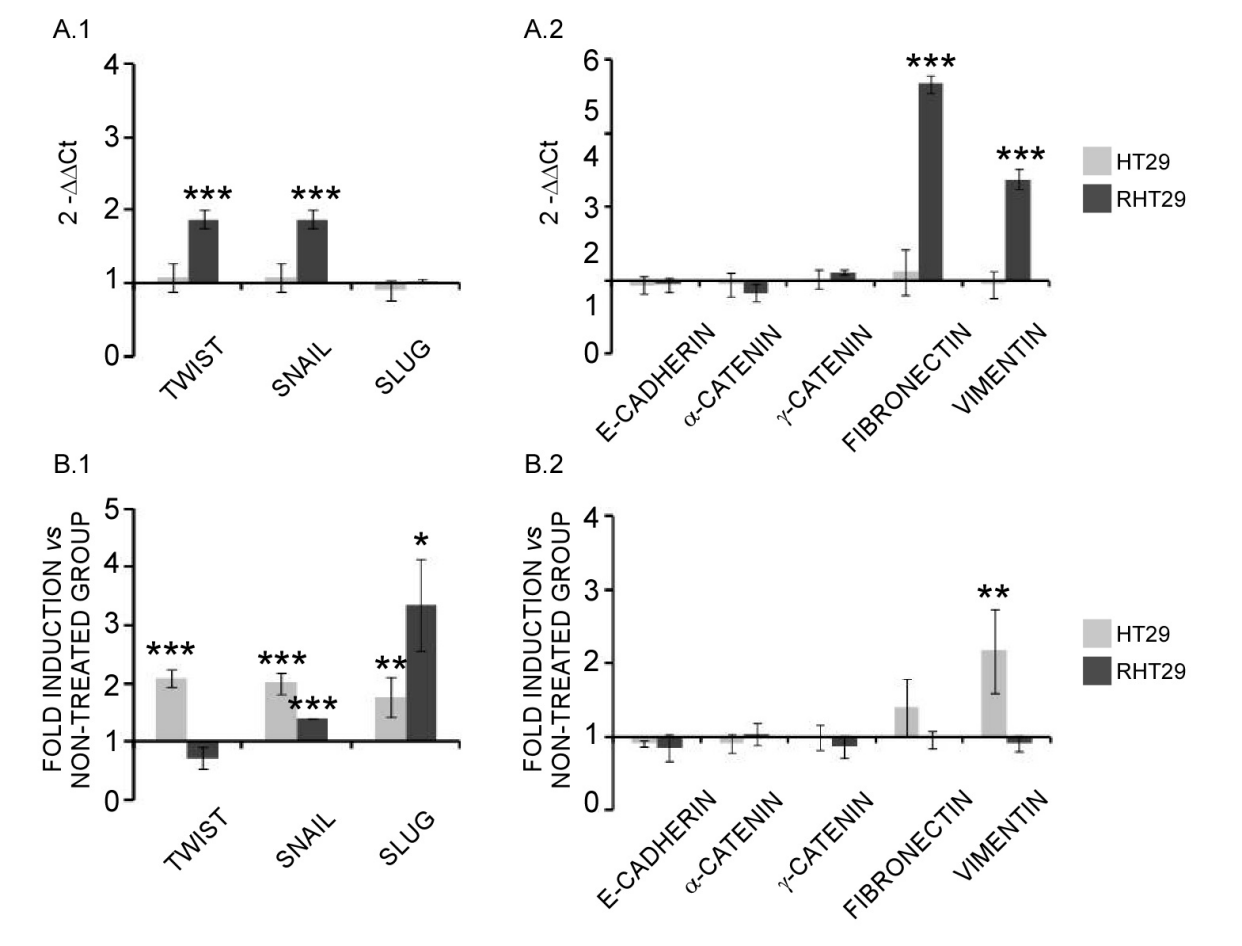
**Analysis of EMT markers on HT29 and RHT29 xenografts**. **(A1) **Differential basal expression of some transcription factors related to the EMT process and **(A2) **markers of epithelial and mesenchymal phenotype were determined by qPCR in tumor xenografts derived from the HT29 and RHT29 cell lines. (**B1**) The effects of oxaliplatin in the gene expression of those transcription factors and (**B2**) EMT markers were also analyzed in xenografted tumors treated with oxaliplatin (i.p. 10 mg/kg) once per week during 27 days and in this case the results are shown as fold induction *versus *non-treated tumors. Results represent the mean ± SEM for 6 xenografts per group. Values that are significantly different between (**A**) groups or between (**B**) non-treated and treated groups by ANOVA's analysis are indicated by *p < 0.05, **p < 0.01, ***p < 0.001.

Finally, in an attempt to explore if this EMT process was also taking place *in vivo*, we analyzed the gene expression of the same markers in xenograft tumors, either treated with oxaliplatin or not, generated from the HT29 and the RHT29 cell lines [14]. Interestingly, similar changes in the basal gene expression pattern of the transcription factors, epithelial and mesenchymal markers related to the EMT process were observed *in vivo *between sensitive and resistant tumors when compared to the *in vitro *data (Figures 5 A1 and 5 A2 and Figure 6 A1 and 6 A2). Furthermore, oxaliplatin treated tumors closely mimic the changes on the expression patterns found *in vitro *(Figures 5 B1 and 5 B2 and Figure 6 B1 and 6 B2), further supporting the ability of oxaliplatin to induce EMT.

Therefore, oxaliplatin resistance induces the acquisition of a mesenchymal phenotype as observed for the RHT29 cell line and its xenograft tumor. Moreover, oxaliplatin treatment seems to induce gene reprogramming and EMT in both sensitive and resistant cells *in vitro *and *in vivo *that specifically confers a more motile phenotype to resistant cells.

## Conclusions

In this study we have used previously characterized cell lines [14] as an *in vitro *model of acquired chemoresistance. We took advantage of the differential CD95 functionality displayed by these cell lines, as a result of the acquired chemoresistance process, to investigate the CD95-mediated cellular responses differentially regulated in sensitive and oxaliplatin-resistant cells. We have focused on the differential effects of oxaliplatin in the activation of MAPK pathways, migration and adhesion and the contribution of CD95 to all these processes, demonstrating that direct or oxaliplatin-induced CD95 activation in chemoresistant cells has tumor-promoting effects. Finally, we have demonstrated that oxaliplatin-treated cells and tumors initiate but do not complete an EMT-like program and that chronic exposure to oxaliplatin renders them more prone to complete EMT, as they had already initiated EMT gene reprogramming during the acquisition of resistance to oxaliplatin. The diverse CD95-mediated oxaliplatin responses described can ultimately be responsible for the different cellular behavior observed between sensitive and resistant cells when exposed to oxaliplatin.

The general assumption that the CD95 receptor works as a death receptor has changed over the last years with the discovery that CD95 can mediate apoptosis-independent processes including proliferation, migration, angiogenesis and inflammation [12,27-29]. These changes in CD95 functionality seem to be related with membrane microdomain location, receptor internalization and endosomal trafficking [30]. However, whether the type of CD95 signaling is an intrinsic trait or a mechanism that can be acquired by changes in cell phenotype has never been addressed before. Moreover, specific tumor-promoting effects of CD95 stimulation in chemoresistant cells have not been reported. In cancer, the responsiveness to CD95 activation has been classified by Scaffidi *et al *[2] as dependent (type II cells) or independent (type I cells) from mitochondria. On the other hand, Algeciras-Schimnich *et al *[3] classified type I cell lines as those corresponding to a mesenchymal phenotype whereas the type II cell lines preferentially express epithelium-like markers.

The results from Algeciras-Schimnich et al [3] suggest that type I and II tumor cells represent different stages of carcinogenesis that resemble the EMT process. In fact, we have observed in our model of acquired chemoresistance that the changes in CD95 signaling are accompanied by EMT, suggesting that CD95 functionality can switch as a consequence of changes in cell phenotype. Moreover, in these chemoresistant cells displaying a mesenchymal phenotype, CD95 signaling strongly induces cell migration and decreases cell adhesion in agreement with the proposed tumor-promoting effects of CD95 in some cell types.

The fact that during the acquisition of chemoresistance CD95 can switch its functionality could have relevant clinical implications. Chemotherapy acts as a selective pressure selecting resistant clones. If chemotherapy fails to eliminate the bulk of the tumor, the resulting resistant clones will sustain the regrowth of the tumor. As demonstrated in this work, these clones selected under chemotherapy show a mesenchymal phenotype and express a CD95 receptor with a non-apoptotic function. Moreover, the continuous exposure of these cells to oxaliplatin will allow the activation of CD95 and the stimulation of tumor-promoting pathways as well as cell migration. Therefore, oxaliplatin treatment of cells resistant to oxaliplatin-induced cell death could ultimately have tumor promoting effects by the upregulation and activation of prosurvival and promigratory CD95-controlled pathways. Then, more research efforts are needed to better understand how the acquisition of chemotherapy is produced, the time point at which tumor cells have acquired resistance to chemotherapy and consistently when the treatment could have detrimental effects.

## Materials and methods

### Cell lines and reagents

Human colon carcinoma cell line HT29 was purchased from American Type Culture Collection (Rockville, MD). The HCT116 p53^-/- ^was generously given by Dr. Vogelstein. The oxaliplatin-resistant cell lines were developed by repeated exposure of the parental cells to increasing concentrations of oxaliplatin as we previously described [14]. Experiments were performed with the resistant population obtained from this process instead of using clones to better mimic the normal process by which a patient develops chemoresistance and to maintain some degree of tumor heterogeneity. All cell lines were cultured in McCoy's Medium supplemented with 10% heat inactivated fetal bovine serum (FBS), antibiotics (Gibco, Invitrogen). The cultures were incubated at 37°C in a humidified 5% CO_2 _atmosphere and the cells were serum starved overnight before experiments.

Antibodies used were mouse monoclonal anti-Fas (clone DX2) from Pharmingen; mouse monoclonal anti-Fas (clone CH11) from Upstate Biotechnology; mouse monoclonal anti-NF-κB (NLS epitope) from Chemicon, rabbit polyclonal antibodies against phospho-p42/44 MAPK, p42/44 MAPK, phospho-p38 MAPK and p38 MAPK, were from Cell Signaling Technology, mouse monoclonal anti-caspase-8 from Calbiochem, mouse monoclonal anti-Bcl2, rabbit monoclonal anti-Bax, mouse monoclonal anti-cyclin D1, rabbit polyclonal antibodies against CDK4, CDK2, and p27 were from Santa Cruz Biotechnology and mouse monoclonal anti-tubulin from Sigma. Oxaliplatin was from Sigma. All general reagents were purchased from Sigma, Bio-Rad and Amersham.

### Western blot

The effects of oxaliplatin in regulatory cell cycle proteins and pro and anti-apoptotic proteins were detected by Western blot in cells treated with oxaliplatin 10 μM during 24 and 48 hours. To study the contribution of CD95 to the induction of MAPK proteins and to the activation of NF-κB the colon cancer cell lines were treated with oxaliplatin (10 μM) for 48 hours and with CH-11 (150 ng/ml) for 24 hours. Protein was extracted and analyzed by Western blot as previously described [31] and tubulin expression was used as endogenous control. Image capture was performed with a Luminiscent Image Analyzer LAS-3000 (Fujifilm, Japan) and image analysis and densitometric quantification by using the Image Reader software (Fujifilm).

### Transfection with antisense CD95

Silencing of CD95 was done by Stealth RNAi technology from Invitrogen as previously described for other genes [14], which provides three different chemical modified oligonucleotides against three different regions of the RNA target. The three probes used for the silencing of CD95 were: Probe 1, Cat#10620318, FASHSS100597: UGACAAUGUCCAAGACACAGCAGAA and Cat#10620319 FASHSS100597: UUCUGCUGUGUCUUGGACAUUGUCA; Probe 2, Cat#10620318 FASHSS100598: GGUUCUUACGUCUGUUGCUAGAUUA and Cat#10620319 FASHSS100598: UAAUCUAGCAACAGACGUAAGAACC; Probe 3, Cat#10620318 FASHSS100599: GGGAUUGGAAUUGAGGAAGACUGUU and Cat#10620319 FASHSS100599: AACAGUCUUCCUCAAUUCCAAUCCC. Before silencing experiments, the protocol was adjusted to ensure a minimum of 80% of RNA knockdown assessed by qPCR.

### Migration and adhesion

Migration assays were performed in transwell inserts (with 8 μm pore size and UV-opaque membrane from Becton Dickinson). The undersurface of transwells were coated with 5 μg of human plasma fibronectin (SIGMA) or 1% BSA (negative control) and blocked with of 2% FBS. Cells previously treated with oxaliplatin oxaliplatin (10 μM for the HT29 and RHT29 and 5 μM for the HCT116 p53^-/- ^and RHTC116 p53^-/-^), CH-11 (150 ng/ml) or or DX2 (400 ng/ml) during 10 hours or silenced for CD95 by siRNA for 48 hours were labeled with Calcein-AM 5 μM (Invitrogen) for 30 min. Then, cells were resuspended in 100 μl of serum-free medium and the corresponding treatment depending on the group (oxaliplatin, CH-11 and DX2), and 130 × 10^3 ^cells per transwell were seeded and allowed to migrate for 14 hours at 37°C (time of every treatment at the end of the experiment was 24 hours). Medium containing a 10% of FBS was placed at the bottom of the wells and used as chemoattractant. After the 14 hours of migration cells on the bottom of the transwell were fluorimetrically detected by using the Biotek SpectraFluor plate reader, with 485 nm excitation and 530 nm emission filters. The experiment was performed in triplicate and fluorescence values were normalized *versus *the non-treated non-stimulated control cells.

For the adhesion assay 24-well plates were coated with fibronectin (50 μg/ml) or 1% BSA (negative control) at 37°C for 3 hours, and blocking of non-specific binding sites was performed using 1% BSA during 30 minutes at 37°C. Before assays, cells were treated with oxaliplatin oxaliplatin (10 μM for the HT29 and RHT29 and 5 μM for the HCT116 p53^-/- ^and RHTC116 p53^-/-^), CH-11 (150 ng/ml) or DX2 (400 ng/ml) depending on the experimental group during 24 hours, or CD95 was silenced by siRNA. Cells were fluorescence-labeled with calcein-AM and 4 × 10^5 ^cells per well were left to adhered on the 24-well coated plates for 2 hours at 37°C. Nonadherent cells were removed by washing the wells with PBS and attached cells were quantified by measuring the fluorescence emission using the Biotek SpectraFluor plate reader, with 485 nm excitation and 530 nm emission filters. Every experiment was performed in triplicate.

### Immunofluorescence Assay

For the detection of E-cadherin cells were seeded in coverslips until subconfluence and treated with oxaliplatin 10 μM for 30 minutes. Then, cells were immunostained as previously described [14] using the mouse primary antibody against E-cadherin (Cell Signaling). Samples were viewed using an inverted epifluorescence microscope (Leica) and image assembly and processing were performed using the Image Processing Leica Confocal Software at the Microscopy Unit of the School of Medicine of the University of Barcelona.

### qPCR Analysis

Total RNA from tumor cells was isolated with Ultraspec (Biotecx) according to the manufacturer's instructions. Quantitative PCR analysis was done on the ABI PRISM 7900 Sequence Detector System (Applied Biosystems) as previously described [14]. For determination of gene expression Assay-on-demand (Applied Biosystems) was used. Transcript levels were normalized to those of beta-actin (Hs99999903_m1), which was used as endogenous control. Each experiment and every determination were done at least in triplicate, and the levels of gene expression were calculated using the ΔΔCt method.

### Cell cycle

Cell cycle analysis was performed as described elsewhere in cells treated with oxaliplatin 10 μM for 24 h. DNA content was determined on a FACscan flow cytometer using FACSCalibur (BD Biosciences) and data processed by the software CellQuest. Every experiment was performed in triplicate.

### *In vivo *experiments

*In vivo *experiments were performed with the HT29 and the RHT29 colon cancer cell lines as previously described [14]. All the experimental procedures with animals were performed in accordance with the regulations of our institution's Ethics Commission, following the guidelines established by the government of Catalonia, Spain.

### Statistical analysis

Statistical analysis of the results was performed by ANOVA analysis. Statistical significance was considered for p values less than 0.05.

## Competing interests

The authors declare that they have no competing interests.

## Authors' contributions

EA and SG performed the experiments, EA and DC did the EMT analysis by qPCR, CM and PF helped in the development of the chemoresistant cell lines, EP gave technical support, EA, NC, VA and PG discussed and interpreted the results, NC, EA and VA wrote the paper.

All authors have read and approved the final manuscript.

## Supplementary Material

Additional file 1 **Figure S1**. Optimization of CD95 silencing by siRNA. Click here for file

Additional file 2 **Figure S2**. Effects of the CD95 blocking antibody DX2 on cell migration and adhesion. Click here for file

Additional file 3 **Figure S3**. Effects of oxaliplatin in cell morphology. Click here for file
