# Supplementary material for: Tumor promoting effects of CD95 signaling in chemoresistant cells
Source: Mol Cancer. 2010 Jun 23;9:161. doi: 10.1186/1476-4598-9-161 (PMC2906471; doi:10.1186/1476-4598-9-161)
Supplement: Additional file 1 — Figure S1. Optimization of CD95 silencing by siRNA. [file 1476-4598-9-161-S1.PDF]

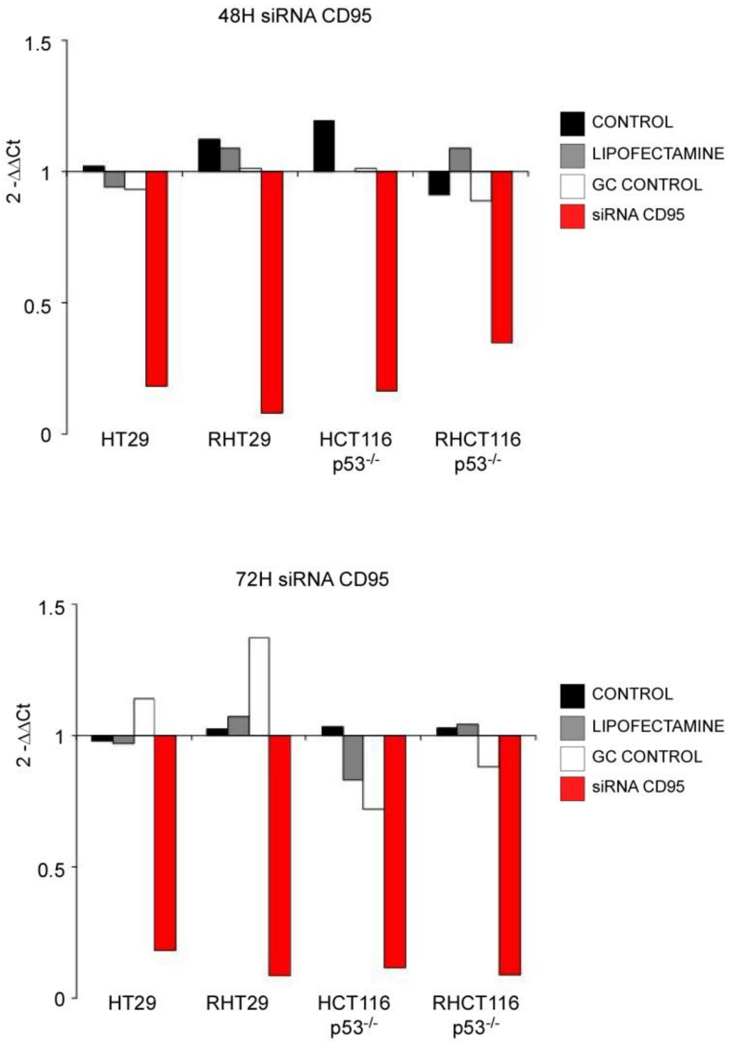

Figure S1. Optimization of CD95 silencing by siRNA. For the silencing of CD95 we used the 3 different synthetic RNAs against different regions of the CD95 mRNA. To confirm the specificity and stability of the silencing over the time we determined the effects of lipofectamine and the GC control (non-specific oligo containing similar GC content than the siRNAs) relative to the effects of the siRNA on CD95 mRNA levels by qPCR at 48 and 72h. The siRNA of CD95 decreased CD95 mRNA levels at least an 80% compared to the controls.
