# Supplementary material for: Tumor promoting effects of CD95 signaling in chemoresistant cells
Source: Mol Cancer. 2010 Jun 23;9:161. doi: 10.1186/1476-4598-9-161 (PMC2906471; doi:10.1186/1476-4598-9-161)
Supplement: Additional file 2 — Figure S2. Effects of the CD95 blocking antibody DX2 on cell migration and adhesion. [file 1476-4598-9-161-S2.PDF]

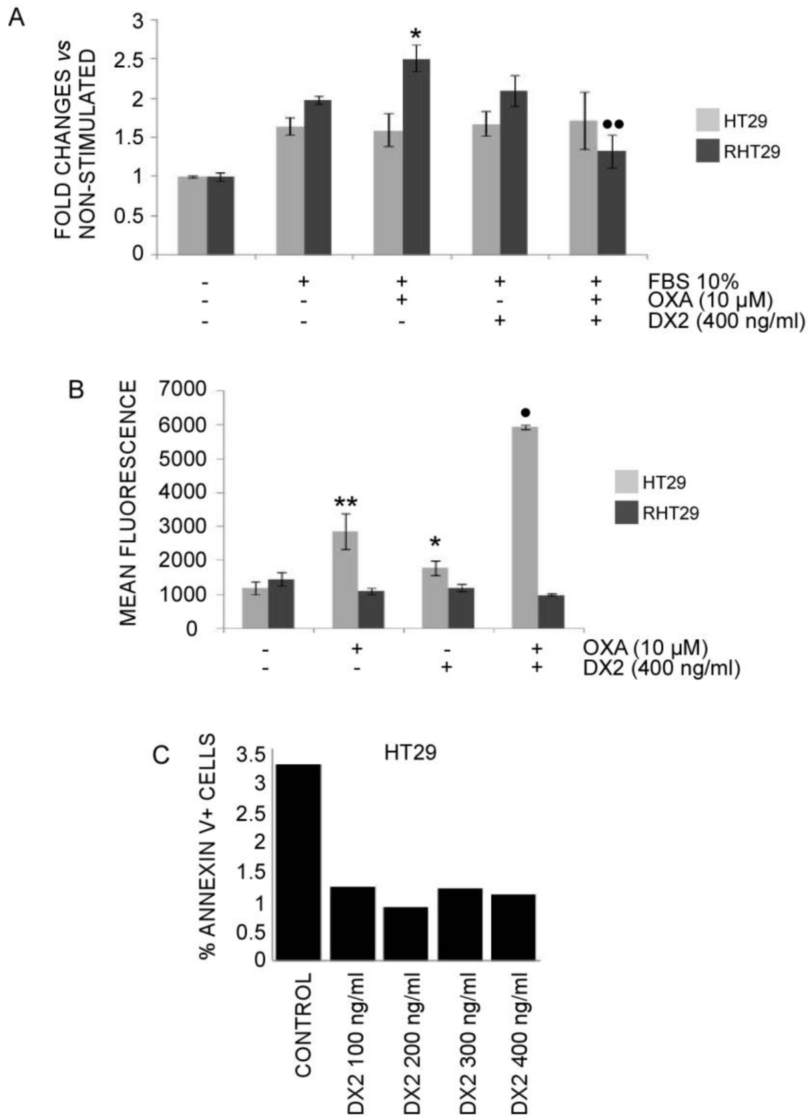

Figure S2. Effects of the CD95 blocking antibody DX2 on cell migration and adhesion. A) Cell migration. **B)** Cell adhesion. **C)** Annexin V binding on the HT29 cell line under increasing DX2 doses. Treatment with DX2 antibody decreased rather than increased the basal levels of annexin positive cells. The experiments were performed in triplicate and results represent the mean±SEM. Values that are significantly different from control group by ANOVA's analysis are indicated by *p<0.05, **p<0.01, and those different from the oxaliplatin-treated group are indicated by •p<0.05, ••p<0.05. *OXA*: oxaliplatin, *FBS*: Fetal bovine serum.
