# Supplementary material for: Tumor promoting effects of CD95 signaling in chemoresistant cells
Source: Mol Cancer. 2010 Jun 23;9:161. doi: 10.1186/1476-4598-9-161 (PMC2906471; doi:10.1186/1476-4598-9-161)
Supplement: Additional file 3 — Figure S3. Effects of oxaliplatin in cell morphology. [file 1476-4598-9-161-S3.pdf]

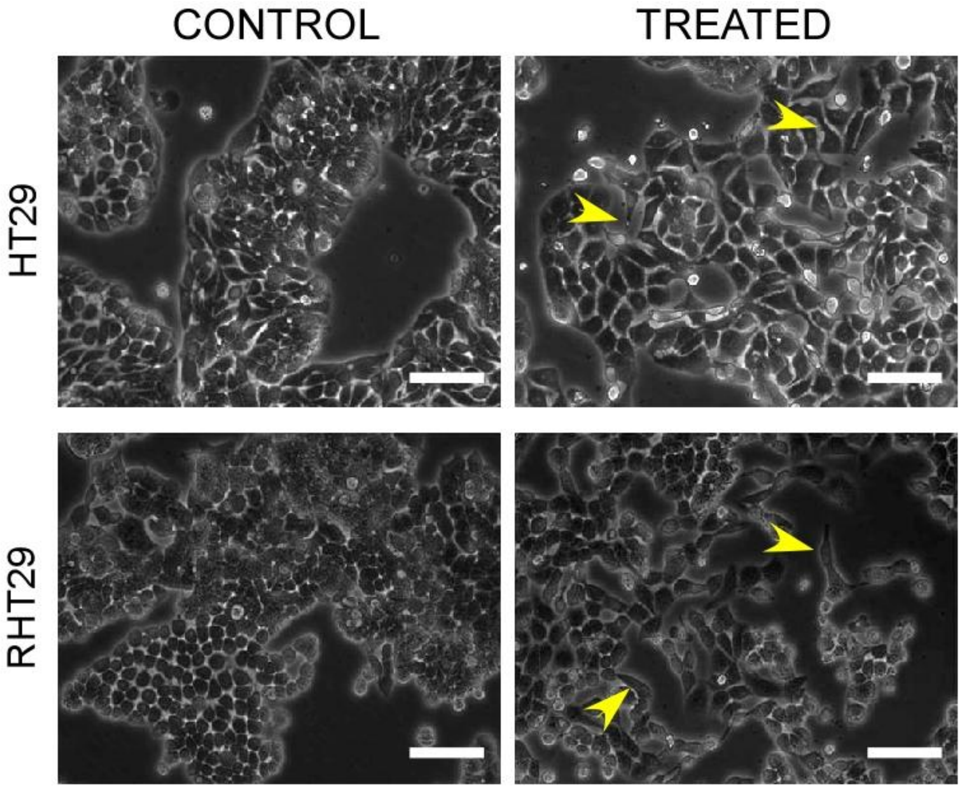

Figure S3. Effects of oxaliplatin in cell morphology. The changes on cell morphology induced by oxaliplatin were analyzed by phase contrast microscopy. After 24 hours of oxaliplatin exposure, both the HT29 and the RHT29 cells suffered a change in cell morphology to a more mesenchymal phenotype. This change in cell morphology was observed in several cells, as indicated by the yellow arrows. Moreover, the cells looked less attached to each other in some regions suggesting that they were losing cell-cell contacts. Scale bar, 50 µm.
